# Deterministic and time resolved thermo-magnetic switching in a nickel nanowire

**DOI:** 10.1038/s41598-019-54043-y

**Published:** 2019-11-22

**Authors:** M. P. Proenca, M. Muñoz, I. Villaverde, A. Migliorini, V. Raposo, L. Lopez-Diaz, E. Martinez, J. L. Prieto

**Affiliations:** 1 Instituto de Sistemas Optoelectrónicos y Microtecnología (ISOM), Universidad Politécnica de Madrid, Avda. Complutense 30, E-28040 Madrid, Spain; 2 IMN-Instituto de Micro y Nanotecnología, (CNM-CSIC), Isaac Newton 8, 28760 Tres Cantos, Madrid Spain; 3 Dpto. Física Aplicada. University of Salamanca, Plaza de los Caídos S/N, E-37008 Salamanca, Spain; 4 IFIMUP and IN-Institute of Nanoscience and Nanotechnology and Dep. Física e Astronomia, Universidade do Porto, Rua do Campo Alegre 687, 4169-007 Porto, Portugal

**Keywords:** Spintronics, Magnetic devices

## Abstract

Heating a ferromagnetic material is often perceived as detrimental for most applications. This is indeed the case for modern nano-scaled spintronic devices which are operated solely (at least ideally) by an electric current. Heat is a by-product of the current-driven operation and it deteriorates many functionalities of the device. A large scientific and technological effort is devoted these days to avoid heat in modern magnetic nano devices. Here we show that heat can be used to provide an additional and useful degree of freedom in the control of the local magnetization at the nanoscale. In a ferromagnetic nanowire, temperature is used to induce a magnetic switching through a perfectly deterministic mechanism. The nucleation of the magnetic domain walls that triggers the switching can be achieved at a field considerably smaller than the nucleation field and, importantly, the exact moment of the magnetic switching can be pre-determined with nanosecond precision by controlling the power delivered locally to the switching area. With the help of micromagnetic simulations and a theoretical model, we provide an accurate explanation of how this deterministic thermo-magnetic switching operates. The concepts described in this work may lead to an increased functionality in magnetic nano-devices based on magnetic domain walls.

## Introduction

Every day we witness reactions that are thermally activated. From the evaporation of water to the denaturalization of the proteins in our fried steak, from diffusion to viscosity, many of the processes and properties that shape our world are thermally activated. If a given reaction has an activation energy *E*_*A*_, the probability for this reaction to happen is proportional to the Boltzmann exponential exp{−*E*_*A*_/*k*_*B*_*T*}, where *k*_*B*_ is the Boltzmann constant and *T* the temperature. This probability is often perceived as the time required for a given process to take place (i.e. if the temperature is too low, the reaction would take too long or it would never happen).

Thermally activated processes are of particular relevance in magnetism. Superparamagnetism is perhaps the most notorious example because of its detrimental effect in magnetic memories^1^. In superparamagnetism, small ferromagnetic particles switch their magnetization direction stochastically due to thermal activation. This process ruins the long term stability of the recorded information in magnetic hard drives and it is becoming a fundamental limit for this type of memories. Alternative proposals for future magnetic memories such as the race-track memory^2^ face also the challenge of the stochastic nature of thermally activated processes. For instance, in materials with perpendicular magnetic anisotropy, the magnetic domain walls (DWs) that define the bits of information can have an unwanted and unpredictable movement activated by heat (creep)^3^. Also in materials with in-plane anisotropy such as Permalloy, the current densities required to operate a race-track memory are so large that the associated Joule heating makes some thermal problems unavoidable^4,5^: unpredictable movement or depinning of the DWs^6,7^, or local loss of the magnetic order^8^. Even in the case of Heat Assisted Magnetic Recording (HAMR)^9^ where heat is intended to have a positive effect (facilitate the writing process), the stochastic nature of a thermally activated process is one of the biggest challenges faced by this technology. The ferromagnetic bit is heated by a focused laser beam close or even above its Curie Temperature (*T*_*C*_) and the collection of grains in the bit, with a distribution in their *T*_*C*_, need to freeze reliably at saturated remanence during rapid cooling^10^. The alignment of the grains is described by superparamagnetism in terms of the temperature and the head field and thus it follows a probability distribution. Achieving a deterministic freezing process when the temperature is close to *T*_*C*_ is not trivial and the topic is in the forefront of research in condensed matter physics.

In the context of the stochastic nature of thermally activated processes in magnetism and the detrimental effect of temperature, here we describe a completely deterministic thermally induced magnetic switching that can be timed with nanosecond precision by controlling the amount of heat delivered to the sample. In a Nickel nanowire, a nanometric section is heated with a pulse of electric current flowing through an adjacent conductive line, altering the local saturation (spontaneous) magnetization due to the temperature increase. The switching is achieved in a deterministic fashion by unbalancing the local demagnetizing field, all in the presence of an external field which is considerably smaller than the Room Temperature switching field. Interestingly, the exact time of switching is temperature dependent and therefore, it can be predetermined with precision of nanoseconds by controlling the power delivered by the current pulse, or sequence of pulses.

## Results

**Figure 1:**
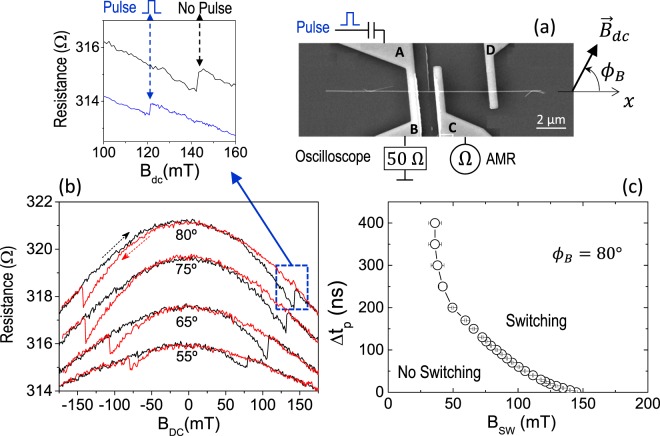
(**a**) SEM photograph of a contacted Ni nanowire (Ni-NW), 80 nm in diameter and 18 µm long. The contacts A, B, C and D are 700 nm wide and 200 nm thick. Contacts C and D are used to monitor the Anisotropic Magnetoresistance (AMR) of the Ni-NW during the magnetization process. Contact A-B is used to apply current pulses that assist the magnetization process in the Ni-NW. **(b)** AMR response of the wire, measured between contacts C and D, for different angles of the external magnetic field (${\overrightarrow{B}}_{dc}$) with respect to the Ni-NW. The curves for each angle are displaced vertically for clarity. The top inset shows the AMR signal without (black line) and with (blue line) the injection of the current pulse along the bit line. **(c)** Switching/No-Switching phase diagram showing the dependence of the switching field (*B*_*SW*_) on the current pulse duration (Δ*t*_*p*_).

Nickel cylindrical nanowires were prepared by electrodeposition and then dispersed on a Si/SiO_2_ (400 nm) substrate. Different nanowires were positioned within the substrate and then contacted with a standard e-beam lithography and lift-off process (see Methods). Figure 1a shows a SEM photograph of a contacted Ni nanowire (Ni-NW), 80 nm in diameter and 18 µm long, which was used for the measurements described in this work. The contacts are 700 nm wide and 200 nm thick with the structure Ta-10nm/ Cu-180nm/ Pt-2nm. Contacts C and D are used to monitor the Anisotropic Magnetoresistance (AMR) of the Ni-NW during the magnetization process. Contact A-B is used to apply current pulses that assist the magnetization process in the Ni-NW and it will be referred in the remaining of the text as the *bit line*. The bit line A-B is electrically isolated from the Ni-NW by a 40 nm thick sputtered SiO_2_ layer.

Figure 1b shows the AMR response of the wire, measured between contacts C and D, for different angles of the external magnetic field (${\overrightarrow{B}}_{dc}$) with the axis of the Ni-NW. The electric current used for this measurement is very small (5–10 µA) and therefore unable to have any contribution in the magnetization process. The abrupt change in resistance observed in the AMR curves, marks the field required to switch the direction of magnetization in the wire, *B*_*SW*_. As we will see in more detail with the micromagnetic simulations, this jump in the AMR signal reflects the difference in the *y*-component of the magnetization, *m*_*y*_, before and after the switching. The change in AMR at *B*_*SW*_ is more visible at higher angles and therefore, we will fix the angle to ${\varphi }_{B}=80^\circ$ for the remaining of this work.

For an external field slightly smaller than *B*_*SW*_, we can trigger the switching of the Ni-NW by injecting a pulse of electric current through the bit line A-B. This is clearly visible in the inset on top of Fig. 1b. The black curve shows the switching for *B*_*SW*_ = 142 mT and no current pulse flowing through the bit line (${I}_{BL}=0$). Then, we repeat the measurement with the following alternative sequence. As before, the external magnetic field is increased in 0.1 mT steps but now, before the next 0.1 mT increment, a single 4 V (*J*_*BL*_ ~ 5·10^11^ A/m^2^; ${I}_{BL}\approx 65\,{\rm{mA}}$) 25 ns pulse is injected through the bit line A-B. As it can be seen in the blue curve of the inset to Fig. 1b, the switching happens now for a field considerably smaller, 121 mT. The amplitude of the pulse, 4 Volts, is the maximum voltage available in the pulse generator. On the other hand, the length of the current pulse (Δ*t*_*p*_) is very influential in the current assisted switching. Figure 1c shows how, as the length of the pulse increases, the switching field *B*_*SW*_ decreases dramatically. The process is deterministic (measured at least 10 times in each point). For instance, if a current pulse of 4 V - 150 ns is injected through the bit line as the external magnetic field is ramped up, the switching will always take place at *B*_*SW*_ = 65 mT. If the current pulse is never injected, the switching will always take place at *B*_*SW*_ = 142 mT. Only for very long pulses ($\Delta {t}_{p}\gtrsim 300\,{\rm{ns}}$) there is uncertainty in the measurement and the transition from ‘no-switching’ to ‘switching’ is not perfectly sharp at a given field.

## Discussion

A possible explanation for the experimental results shown in Fig. 1 is to assume that the current pulse is assisting the switching of the Ni-NW through its associated Oersted field (${\overrightarrow{B}}_{Oe}$) which, in addition to the external magnetic field (${\overrightarrow{B}}_{dc}$), may reach (locally) the correct value of the switching field (142 mT). However, this would not explain why the length of the current pulse is such a relevant parameter in the switching field (Fig. 1c). As the amplitude of the pulse is always the same (${I}_{BL}=65\,{\rm{mA}}$), the associated Oersted field should be constant, and the length of the pulse should have marginal or no influence, which is clearly not the case. This is confirmed by zero temperature micromagnetic (µM) simulations.

**Figure 2:**
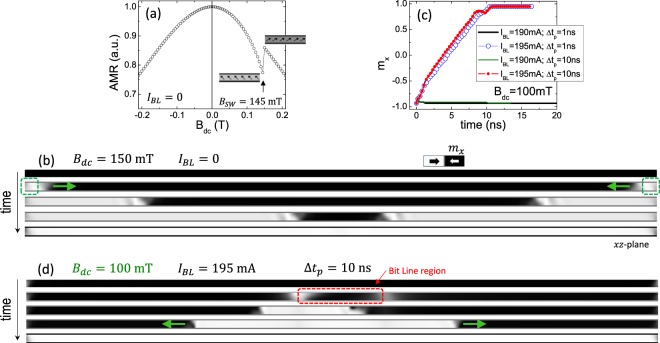
(**a**) Anisotropic Magnetoresistance (AMR) versus the external field in the absence of current pulses along the bit line at room temperature. The two snapshots at 145 mT show the small difference in the y-component of the magnetization before and after the jump **(b)** Micromagnetic snapshots of the magnetization in the central plane of the Ni-NW for an initial state along the negative *x*-axis under an external field of ${B}_{dc}=150\,{\rm{mT}}$ at ${\varphi }_{B}=80^\circ$. No current pulse is applied along the bit line. **(c)** Temporal evolution of the averaged Ni-NW magnetization along the *x*-axis for different combinations of *B*_*dc*_ and current pulses along the bit line with different amplitudes (*I*_*BL*_) and durations (Δ*t*_*p*_). **(d)** Transient micromagnetic snapshots for ${B}_{dc}=100\,{\rm{mT}}$, ${I}_{BL}=195\,{\rm{mA}}$ and Δ*t*_*p*_ = 10 ns. These results were obtained at zero temperature by solving the LLG eq (S1).

First, in Fig. 2a we reproduce with our µM-simulations the experimental AMR loop in the absence of current pulses (${I}_{BL}=0$). The switching field obtained is ${B}_{SW}^{\mu M}=145\,{\rm{mT}}$, very similar to the experimental value. Therefore, µM-simulation captures well the physics involved in the experimental AMR loop. Figure 2b shows the transient magnetization snapshots in the central plane of the Ni-NW under an external field of ${B}_{SW}^{\mu M}=150\,{\rm{mT}}$. As it can be seen, in absence of current pulses, the switching takes place by the nucleation of two domain walls (DWs), one at each end of the Ni-NW. These DWs are driven by the static field towards the centre of the Ni-NW, where they annihilate completing the magnetization reversal.

With this zero temperature µM-simulation we can also explore the conditions for switching taking into account the Oersted field generated by a current pulse. In the absence of external field (${B}_{dc}=0$), the minimum current to nucleate a reversed domain below the bit line is ${I}_{BL}\approx 275\,{\rm{mA}}$ (see S1.c in Supplementary Information), which is almost four times larger than the experimental value. Interestingly, no matter how large the value of the current or the length of the pulse (Δ*t*_*p*_) is, for ${B}_{dc}=0$, the pair of nucleated DWs will travel towards the centre of the nanowire and collapse, so the switching of the Ni-NW is never completed (see snapshots in Fig. S3 in Supplementary Information).

When there is a non-zero external field (*B*_*dc*_) smaller than ${B}_{SW}=142\,{\rm{mT}}$, the reversed domain can be nucleated only if the amplitude of the current pulse is significantly larger than the experimental one. For instance, if ${B}_{dc}=100\,{\rm{mT}}$, a minimum amplitude of ${I}_{BL}=195\,{\rm{mA}}$ is needed to achieve the Ni-NW reversal, as it is shown in Fig. 2c. In this case, the local nucleation takes place during the first l ns, and the two DWs that flank the reversed domain, rapidly propagate in opposite directions completing the switching due to the longitudinal component of the external field, as shown in the snapshots in Fig. 2d.

As a resume of the zero temperature µM-simulations, only if the external field and the amplitude of the current pulse are sufficiently large, the switching of the Ni-NW is always achieved, but irrespective of the length of the current pulse and for pulse amplitudes considerably larger than the experimental value. It was also confirmed that the scenario described in Fig. 2 does not change if the Ni-NW is at a non-zero uniform temperature ($T=300\,{\rm{K}}$) or by adding defects or pinning sites in the Ni-NW (see Supplementary Information S1.d). Therefore, if the temperature of the Ni-NW is uniform and constant, the µM-simulations cannot be reconciled with the experimental results, and it is not possible to make the switching field (*B*_*SW*_) dependent on the length of the current pulse (Δ*t*_*p*_) as the experiments show in Fig. 1c.

Clearly, the current pulse must be contributing to the switching of the Ni-NW not only with the associated Oersted field (${\overrightarrow{B}}_{Oe}$) but also with an additional source of energy, which predictably is going to be heat. As the current density flowing through the bit line during the pulse is quite relevant (*J*_*BL*_ ~ 5·10^11^ A/m^2^) and the substrate has a thick (400 nm) SiO_2_ layer with poor thermal conductivity, the associated Joule heating is not going to be negligible. This heat will be conducted to the Ni-NW underneath and, as we will see, ultimately determine the value of the switching field *B*_*SW*_.

In order to evaluate the temperature along the Ni-NW we have performed COMSOL^11^ simulations with the exact geometry of the experimental set up. In advance of the thermal characterization that we are about to describe, it should be noted that only a very precise prediction of the temperature distribution in the Ni-NW could explain and fit the experimental results shown in Fig. 1c. Therefore, the material properties pumped into the COMSOL simulation were obtained either experimentally from the same device used for the experimental results in Fig. 1, or from the literature for very similar geometries. The most important material properties used here are the electric resistivity and the thermal conductivity of the Copper in the bit line: *ρ*_*Cu*_ = 4·10^−8^ Ω·m and *κ*_*Cu*_ = 165 Wm^−1^K^−1^ respectively, and the thermal conductivity of the Ni-NW, *κ*_*Ni*_ = 22 Wm^−1^K^−1^. Refer to Sec. S2 in Supplementary Information for a detailed explanation of why these properties are appreciably different to the corresponding bulk values. The rest of the parameters were extracted from the COMSOL library and, in any case, they are not so crucial for the results of the thermal simulation. A very important aspect to keep in mind is that the Ni-NW cannot dissipate heat to the substrate. As the Ni-NWs are dispersed on top of the substrate, there is only a thin organic layer between the nanowire and the substrate that does not conduct appreciable heat. The heat that enters the Ni-NW from the bit line A-B can only be dissipated through the Copper contact pads and through the 40 nm thick SiO_2_ window deposited on top of the Ni-NW to isolate it electrically from the bit line A-B.

**Figure 3:**
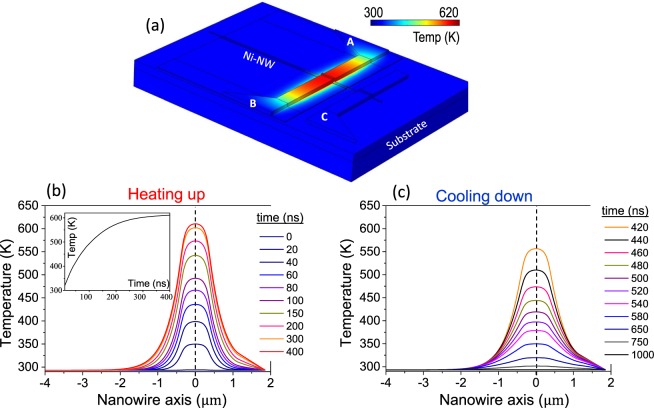
**(a)** Device geometry showing the local temperature after a current pulse of Δ*t*_*p*_ = 400 ns. **(b)** Temperature along the Ni-NW for different instant of time during the heating up (*t* < Δ*t*_*p*_ = 400 ns). The inset shows the evolution with time of the temperature of the hottest point in the Ni-NW **(c)** Temperature along the Ni-NW for different instant of time during the cooling down (*t* < Δ*t*_*p*_ = 400 ns).

Figure 3a shows colour coded cartoon of the COMSOL model with the local temperature after a 400 ns pulse. The centre of the bit line is the hottest point and this heat is transmitted to the Ni-NW, which diffuses laterally until it is dissipated by the SiO_2_ window on top or by the Cu contact pad C. Figure 3b shows the temperature along the Ni-NW axis at different times of the 400 ns current pulse. The temperature at position *x* = 0 (underneath the centre of the bit line) evolves with time as shown in the inset to Fig. 3b, following the expression $\,T(t)\approx 620\,{\rm{K}}-327\,{\rm{K}}\,\exp (-t/\tau )$, with $\tau =104$ ns. At the end of a 400 ns pulse, the region of the Ni-NW underneath the bit line is very close to its *T*_*C*_ (631 K for bulk Ni).

**Figure 4:**
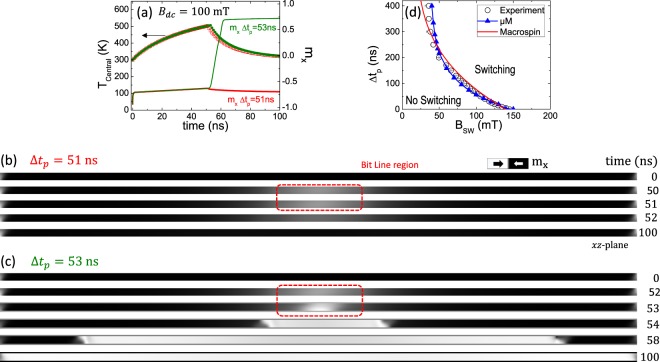
(**a)** Temporal evolution of the temperature in the centre of the Ni-NW just below the current line (${T}_{Central}\,vs\,time$), and temporal evolution of the normalized averaged magnetization in the NW axis (${m}_{x}\equiv {m}_{x}\,vs\,time$) for two pulse durations (Δ*t*_*p*_ = 51 ns and 50 ns) with *B*_*dc*_ = 100 mT. The amplitude of the current pulse is *I*_*BL*_ = 65 mA, i.e., the same amplitude as in the experimental results presented in Fig. 1. **(b,c)** some transient micromagnetic snapshots showing the local magnetization in the Ni-NW for the two current pulses of (a). **(d)** Switching/No-Switching phase diagram showing the dependence of the current pulse duration (Δ*t*_*p*_) on the switching field *B*_*SW*_. Open circles correspond to the experimental results of Fig. 1(c). Blue triangles are micromagnetic results, solid red line corresponds to the predictions of the Macrospin model. The micromagnetic results were obtained by solving the magneto-thermal problem as described in the Supplementary Information S3.

Looking at the results of the thermal characterization in Fig. 3, it becomes clear that the µM-simulations shown in Fig. 2 could not provide a realistic description of the experimental switching process because they did not include the considerable temperature reached locally in the Ni-NW. We cannot use the Landau-Lifshitz-Gilbert equation, which is only valid for a uniform temperature well below the Curie threshold. The magnetization dynamics need to be studied using the Landau-Lifshitz-Bloch equation^12^, which is numerically solved coupled to the heat transport equation (see Sec. S3 in Supplementary Information). Note that within this magneto-thermal framework, the magnetization dynamics takes into account the temperature dependence of the magnetic parameters, such as ${M}_{s}={M}_{s}(T)$ or $A=A(T)$. The results of this µM-simulations using the Landau-Lifshitz-Bloch eq. are summarized in Fig. 4. Figure 4a shows two representative cases where the amplitude of the current pulse is fixed to the experimental value ${I}_{BL}=65\,{\rm{mA}}$. Figure 4a depicts the temporal evolution of the temperature in the centre of the Ni-NW just below the bit line (${T}_{Central}\,vs\,time$, left vertical axis), along with the temporal evolution of the averaged magnetization along the Ni-NW axis (${m}_{x}\equiv {m}_{x}\,vs\,time$, right vertical axis) for two representative combinations of pulse durations (Δ*t*_*p*_ = 51 ns and 53 ns) with a fixed external field ${B}_{dc}=100\,{\rm{mT}}$. For Δ*t*_*p*_ = 51 ns the switching is not achieved as it becomes clear by looking at the corresponding *m*_*x*_ *vs time* (red curve), where *m*_*x*_ remains always negative. The 51 ns pulse heats up the local area of the Ni-NW to a maximum of 503 K and, when the current pulse is turned off (*t* > Δ*t*_*p*_), the Ni-NW starts to cool down and the nucleation of a reversed domain below the bit line is never achieved. The high temperature implies a local reduction of the saturation magnetization (*M*_*s*_ = *M*_*s*_(*T*)) in the section below the bit line, visible by the grey colour in the corresponding snapshots in Fig. 4b.

The situation is completely different for the slightly longer Δ*t*_*p*_ = 53 ns ns pulse. In this case, the maximum temperature reached is only 4 K hotter than in the previous case, but now the switching is achieved and, just after 53 ns, ${m}_{x}\equiv {m}_{x}$ quickly evolves from negative to positive values (green solid line in Fig. 4a). Note that the central temperature increases with Δ*t*_*p*_, and consequently the local saturation magnetization (*M*_*s*_) also decreases with Δ*t*_*p*_. The reversed domain nucleates at $t=53\,{\rm{ns}}$ and it grows rapidly by propagation of the two DWs in opposite directions due to the external field *B*_*dc*_, as shown in the snapshots of Fig. 4c. Additional movies describing these processes are also provided as supporting Supplementary Material. Therefore, a small increment in the duration of the current pulse results in a small increase of the local temperature, but in an incremental reduction of the local saturation magnetization that seems to be crucial for the nucleation of the DWs and the subsequent switching. It is important to mention that, despite heat being the key ingredient in the switching process, µM-simulations show that this switching is deterministic, *i.e*., similar micromagnetic results were obtained in the absence of any stochastic thermal field (See Fig. S9 in Sec. 4 of Supplementary Information). This is also in agreement with the experimental results of Fig. 1c. It is also important to notice that the heat significantly modifies the reversal process with respect to the Oersted field-induced switching shown in Fig. 2d. Now, at the central part of the Ni-NW, the local temperature of the Ni-NW reaches its maximum value, and the associated reduction of the saturation magnetization triggers the switching in a deterministic fashion.

Once the key points of the magneto-thermal dynamics have been described taking into account the local heating, we now numerically evaluate the minimum pulse duration (Δ*t*_*p*_) required to switch the Ni-NW as a function of the applied field (*B*_*dc*_). The micromagnetic results are shown in Fig. 4d. Note that here the amplitude of the current pulse used for the simulation is the same than the one used in the experiment, ${I}_{BL}=65\,{\rm{mA}}$, which is significantly smaller than the current required to achieve a reversed domain for a constant temperature in the Ni-NW (zero or uniform room temperature) as discussed in Fig. 2. In Fig. S9 of the Supplementary Information we provide additional evidences supporting our conclusions on the key role played by the local heat in these processes. In particular, Fig. S9b indicate the dominant role of the heat with respect to the marginal role of the Oersted field (${\overrightarrow{B}}_{Oe}$) associated to the current pulse, which only becomes noticeable as *B*_*dc*_ decreases. Moreover, switching/no-switching transition is found to be independent on the Gilbert damping (see Fig. S9c), a fact that stresses even further the dominant role of heat as compared to any other magnetic dynamics phenomena. Finally, µM-simulations presented in Fig. S9c also reflects the minor role of thermal fluctuations, which supports the deterministic nature of these heat controlled switching processes.

The µM-simulations have shown that the experimental results cannot be explained in a framework of constant temperature in the Ni-NW. Only when local heating is added into the µM-simulations by solving the Landau-Lifshitz-Bloch equation coupled to the heat transport equation, the model could fit the experiment. Still, it is difficult to grasp why the heat and the reduction of the local saturation magnetization make the switching mechanism deterministic. If the magnetic switching described in this work were a consequence of a standard thermally activated process, the Ni-NW (or a section of it) would switch with a probability proportional to $\exp \{-{\epsilon }(H)/{k}_{B}T\}$, being ${\epsilon }(H)$ the energy barrier that the magnetization would need to overcome to switch the magnetization direction. This mechanism would be stochastic and it does not fit with the deterministic switching described by the experiment or by the µM-simulations.

**Figure 5:**
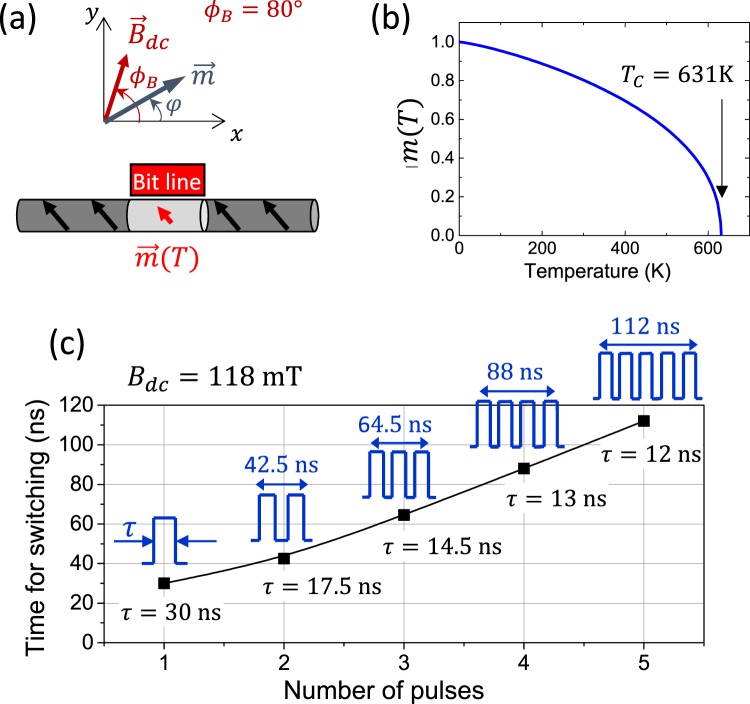
(**a)** Schematic representation of the Ni-NW where a section is heated and its saturation magnetization is reduced as shown by the Langevin function represented graphically in **(b). (c)** Demonstration of the time resolved deterministic switching. In this experiment, we have pre-determined the exact moment of switching by selecting a train of pulses and adjusting the Time-On (*τ*) of each pulse and Time-Off after each pulse. *τ* (the length of each individual pulse) and the entire length of the train of pulses is indicated next to each experimental point.

In order to clarify the thermal deterministic switching mechanism in our system, we put forward a simple macrospin model schematically represented in Fig. 5a. As the temperature increases due to the current pulse, the local saturation magnetization in the hot section of Ni-NW decreases creating a region with smaller saturation magnetization than in the rest of the nanowire. The model assumes that this hot region is uniformly magnetized and that it reverses independently of the rest of the nanowire. Based on these assumptions, we account for the magnetostatic energy of the hot region and obtain from it an expression for the switching field. This switching field is a monotonically decreasing function of temperature through the temperature dependent saturation magnetization *M*_*s*_(*T*). Thus, for each value of the external field *B*_*dc*_, we compute the instant in time that makes ${B}_{sw}(T(t))={B}_{dc}$. The results are shown in Fig. 4d (red curve) together with experimental data and micromagnetic simulations. More details of how this curve is obtained are given in section S6 of Supplementary Information.

The µM-simulations together with the macrospin model show how the heat flow in our system modifies the reversal mechanism from localized nucleation, critically dependent on disorder and stochastic in nature, towards an almost coherent rotation of a well-defined volume in the central region, hardly dependent on disorder and completely deterministic.

Beyond the fundamental interest of describing a mechanism that leads to a deterministic thermal switching, from the practical point of view this process allows to pre-determine the exact time of switching by controlling the power delivered to the nanowire. This can be done with a single pulse by tuning both the amplitude and duration of the pulse or alternatively, with a train of pulses by playing with the on-off times. This is shown in Fig. 5c. In this experiment, the external magnetic field is fixed to *B*_*dc*_ = 118 mT. For this field, a single 4 V pulse (*J*_*BL*_ ~ 5·10^11^ A/m^2^) 30 ns long, generates enough local heat to induce the switching. Alternatively, we can deliver different sequences of pulses to delay the nucleation of the domain walls. For instance, a sequence of 3 pulses, each 14.5 ns long, spaced 10.5 ns, delays the switching to 60 ns. The experiment described in Fig. 5c is a clear example of the true potential of this mechanism. This thermal switching allows the nucleation of the domain wall at a smaller field than the room temperature nucleation field and, at the same time, the exact time of nucleation can be chosen deterministically. This could add functionality to a magnetic memory or to any of the many spintronic devices based on DWs. For instance, one could achieve a perfectly timed nucleation at an external field of choice, small enough to leave unaffected other DWs that may be present in the magnetic device. Note that this experiment should also be possible on a standard nanostrip geometry, which perhaps poses less fabrication challenges than the experiment we present in this work. We hope this deterministic thermal switching can help the future development of new concepts and spintronic devices.

## Methods

### Fabricating and contacting the nickel nanowires

Cylindrical nickel nanowires, with a nominal length of about 38 µm, were electrodeposited inside a nanoporous alumina template with pore diameters and interpore distances of around 80 and 105 nm, respectively^13,14^. To access individual NWs, the templates were chemically etched in 1 M NaOH and the wires rinsed with ethanol 99.5%. The NWs were then dispersed on top of a Si/SiO_2_ substrate with pre-patterned gold pads to allow precise location of the individual nanowires. Pre-patterned gold pads where also used as reference marks to align the contacts and current lines with the selected Ni-NW. After positioning the different nanowires with respect to the reference marks, the contact pads were designed and deposited by a standard e-beam lithography lift-off process.

## Supplementary information

Supplementary Information

Non Switching Event

Switching Event

## References

[1] Moser A (2002). Magnetic recording: advancing into the future. J. Phys D: Appl. Phys..

[2] Parkin SSP, Hayashi M, Thomas L (2008). Magnetic domain-wall racetrack memory. Science.

[3] Metaxas PJ (2007). Creep and Flow Regimes of Magnetic Domain-Wall Motion in Ultrathin Pt/Co/Pt Films with Perpendicular Anisotropy. Phys. Rev. Lett..

[4] Yamaguchi A, Hirohata A, Ono T, Miyajima H (2012). Temperature estimation in a ferromagnetic Fe–Ni nanowire involving a current-driven domain wall motion. J. Phys.: Condens. Matter.

[5] Ramos. E, López C, Akerman J, Muñoz M, Prieto JL (2015). Joule heating in ferromagnetic nanostripes with a notch. Phys. Rev. B..

[6] Torrejon J (2012). Unidirectional Thermal Effects in Current-Induced Domain Wall Motion. Phys. Rev. Lett..

[7] Raposo V, Moretti M, Hernandez MA, Martinez E (2016). Domain wall dynamics along curved strips under current pulses: The influence of Joule heating. Appl. Phys. Lett..

[8] Moretti S, Raposo S, Martinez V (2016). E. Influence of Joule heating on current induced domain wall depinning. J. Appl. Phys..

[9] Kryder MH (2008). Heat Assisted Magnetic Recording. Proc. IEEE.

[10] Ju G (2015). High Density Heat-Assisted Magnetic Recording Media and Advanced Characterization-Progress and Challenges. IEEE Trans. Mag..

[11] COMSOL Multiphysics®. Version 5.4 (Build 225). COMSOL, Inc., Burlington, MA, USA.

[12] Moretti S, Raposo V, Martinez E, Lopez-Díaz L (2017). Domain wall motion by localized temperature gradients. Phys. Rev. B.

[13] Proenca MP, Sousa CT, Ventura J, Vazquez M, Araujo JP (2012). Ni growth inside ordered arrays of alumina nanopores: Enhancing the deposition rate. Electrochim. Acta.

[14] Sousa CT (2014). Nanoporous alumina as templates for multifunctional applications. Appl. Phys. Rev..

