# Supplementary material for: Deterministic and time resolved thermo-magnetic switching in a nickel nanowire
Source: Sci Rep. 2019 Nov 22;9:17339. doi: 10.1038/s41598-019-54043-y (PMC6874679; doi:10.1038/s41598-019-54043-y)
Supplement: Supplementary file 1 — Supplementary Information [file 41598_2019_54043_MOESM1_ESM.pdf]

Supplementary Information:
Deterministic and time resolved thermo-magnetic switching in a nickel nanowire

M. P. Proenca[1, 4], M. Muñoz[2], I. Villaverde[1], A. Migliorini[1], V. Raposo[3], L.Lopez-Diaz[3], E. Martinez[3], J. L. Prieto[1]

[1]Instituto de Sistemas Optoelectrónicos y Microtecnología (ISOM), Universidad Politécnica de Madrid, Avda. Complutense s/n, E-28040 Madrid, Spain.

[2]IMN-Instituto de Micro y Nanotecnología, (CNM-CSIC), Isaac Newton 8, 28760 Tres Cantos, Madrid, Spain

[3]Dpto. Física Aplicada. University of Salamanca, Plaza de los Caídos S/N, E-37008, Salamanca, Spain.

[4] IFIMUP and IN-Institute of Nanoscience and Nanotechnology and Dep. Física e Astronomia, Universidade do Porto, Rua do Campo Alegre 687, 4169-007 Porto, Portugal

<u>Index</u>

S1. Micromagnetic simulations in the absence of heat

S1.a. Hysteresis loops in the absence of current pulses flowing through the bit line

S1.b. Magnetization dynamics under static external magnetic field and current pulses

S1.c. Nucleation in the absence of external field

S1.d. The role of the Gilbert damping and thermal fluctuations for uniform temperature

S2. Heat transport characterization by COMSOL simulations

S3. Details of micromagnetic simulations with Joule heating

S4. Micromagnetic results with Joule heating

S5. Supporting Material: videos of the magneto-thermal dynamics

S6. Details of macrospin model

S1. Micromagnetic simulations in the absence of heat

In order to support our interpretation of the experimental results, we firstly performed conventional micromagnetic simulations in the absence of Joule heating. This section is devoted to present details of this modelling and additional results supporting our claims on the impossibility of explaining the experimental results without considering a non-uniform heat in the nanowire.

The geometry simulated mimics the experimental one, and it is shown in Fig. S1(a). It consists on a Ni nanowire (Ni-NW) along the x-axis. Its length and radius are ℓ and a respectively. The bit line (contact A-B in Fig. 1(a)) has rectangular cross section $w_{BL} \times t_{BL}$ and it is placed on top and orthogonal to the Ni-NW. The vertical distance (along the z-axis) between the bit line and the Ni-NW is s. The dimensions of the Ni-NW and the bit line are: $\ell = 5120$ nm, $a = 40$ nm, $w_{BL} \times t_{BL} = 700$ nm $\times$ 200 nm, and $s = 40$ nm. All dimensions are exactly the same as in the experiment except for the length of the Ni-NW, which is fixed to $\ell = 5120$ nm to save computational effort.

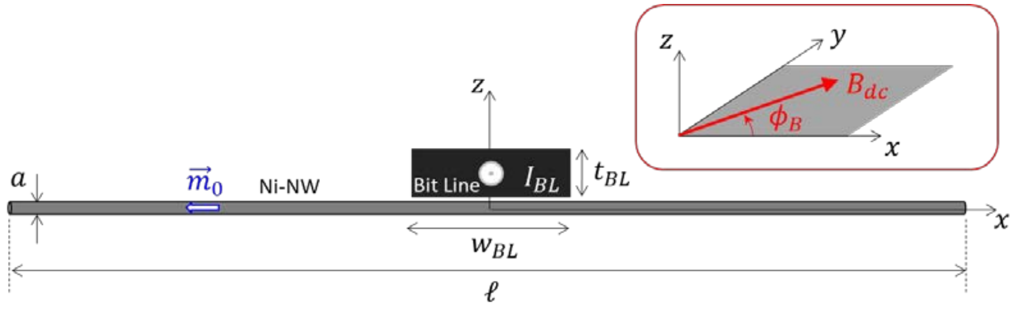

Fig. S1. Geometry of the system studied micromagnetically (xz plane**)**. The values of the defined dimensions are given in the text. A static magnetic field $\vec{B}_{dc}$ is applied during and after the injection of the current pulses through the bit line. The inset indicates the magnitude and direction of the external magnetic field. The current pulses injected through the bit line (along the y-axis) have a magnitude of I_{BL} and a duration of Δt_{BL}. $\vec{m}_0$ represents the initial state of the normalized magnetization ($\vec{m}_0 = \vec{M}(t=0)/M_s$) prior to the application of the current pulse and the static field. It points along the negative direction of longitudinal axis of the Ni-NW, i.e. $\vec{m}_0 = -\vec{u}_x$.

S1.a. Hysteresis loops in the absence of current pulses flowing through the bit line

Before analysing the magnetization dynamics under both current pulses ($I_{BL}, \Delta t_p$) and the static magnetic field $\vec{B}_{dc}$ at zero temperature, we studied micromagnetically the switching without any current pulse flowing through the bit line ($I_{BL} = 0$). Starting from the saturated state along a negative direction we compute the equilibrium state for a sequence of external fields $\vec{B}_{dc}$ (with $\theta_B = 90°$ and $\phi_B = 80°$) of increasing magnitude, from $|\vec{B}_{dc}| = -350$ mT to $|\vec{B}_{dc}| = +350$ mT. The following parameters were adopted, which are typical of Ni: saturation magnetization $M_s = 4.9 \times 10^5$ A/m, exchange constant $A_{ex} = 0.9$ pJ/m and a Gilbert damping parameter $\alpha = 0.1$. Each equilibrium state is numerically computed using MuMax3 by minimizing the total energy of the system [**S1**], which includes exchange ($\vec{H}_{exc}$), magnetostatic ($\vec{H}_{dmg}$) and Zeeman ($\vec{H}_{dc}$) interactions. The Ni-NW was discretized in cubic cells of $\Delta x = 2$ nm. The micromagnetic results are shown in Fig. 2(a) of the main text, which are in good quantitative agreement with the experimental results. The micromagnetically computed zero-current switching field is $B_{SW} = 145$ mT, which is similar to the experimental value ($B_{SW} = 142$ mT). The AMR signal defined as AMR $= \frac{1}{2}(1 - m_x^2)$ with $m_x \equiv \langle m_x \rangle$ being the average longitudinal magnetization along the Ni-NW, looks similar to the experimental one (see Fig. 1(b) for $\phi_B = 80^0$). This indicates that the material parameters adopted in the micromagnetic simulation are appropriate to describe numerically the experimental sample. Without any current pulses delivered through the bit line, the switching takes place by nucleation of two domain walls (DWs) at the two ends of the Ni-NW which propagate in opposite direction along the longitudinal x-axis, and annihilate in the center completing the full reversal of the magnetization (see Fig. 2(b) in the main text).

S1.b. Magnetization dynamics under static external magnetic field and current pulses

Here we describe the micromagnetic details of magnetization dynamics under external magnetic field ($\vec{B}_{dc}$) and current pulses delivered through the bit line. The current pulses have a magnitude $I_{BL} = J_{BL} w_{BL} t_{BL}$ and a duration Δt_p. Current pulses are considered to have zero

rise and fall times. $\vec{m}_0$ represents the initial state of the normalized magnetization ($\vec{m}_0 = \vec{M}(t=0)/M_s$) prior application of the current pulse, and its value is $\vec{m}_0 = -\vec{u}_x$.

The current pulse flowing through the bit line generates an Oersted field $\vec{B}_{Oe} = \mu_0 \vec{H}_{Oe}$ which influences on the magnetization dynamics. This magnetic field was numerically computed in all points of the Ni-NW **[S2].** Its spatial dependence along the Ni-NW axis (x-axis) is shown in Fig. S2. The longitudinal component ($B_{Oe,x}$, left graph in Fig. S2) has a positive magnitude below the bit line, and therefore, it opposes to the initial magnetic state ($\vec{m}_0 = -\vec{u}_x$). On the other hand, the perpendicular component ($B_{Oe,z}$, left graph in Fig. S2) has positive and negative values at the left and right sides of the Ni-NW, respectively. Note that the z-component of $\vec{B}_{Oe}$ is significantly stronger than the x-component, in particular, at the side edges of the bit line ($x = \pm\frac{w_{BL}}{2} = \pm 350$ nm).

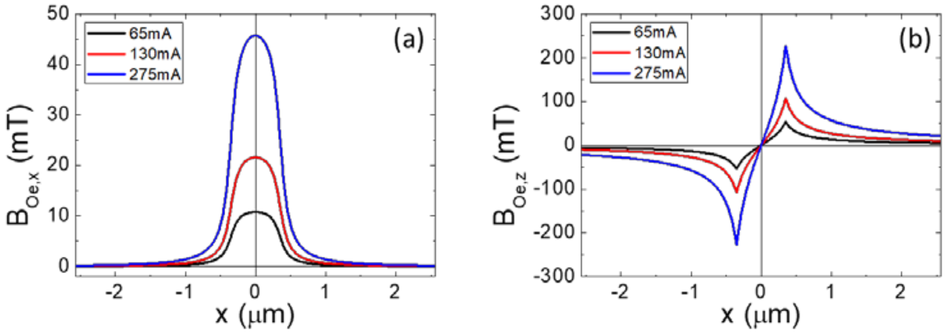

Fig. S2. Oersted field generated by current pulses along the bit line along the Ni-NW axis. **(a)** and **(b)** graphs show the x and z components of the $\vec{B}_{Oe}$ respectively for different values of current amplitude I_{BL}. Note that the experimental amplitude is $I_{BL} = 65$ mA.

Once described the Oersted field due to the current pulse along the bit line, now we focus our attention on the magnetization dynamics. Firstly, we assumed zero temperature ($T = 0$), and consequently we are neglecting any local heating generated by the current pulse. This was done by solving the Landau-Lifshitz-Gilbert eq.

$$\frac{d\vec{m}}{dt} = -\gamma_0 \vec{m} \times \left(\vec{H}_{eff} + \vec{H}_{th}\right) + \alpha \vec{m} \times \frac{d\vec{m}}{dt} \quad \text{(S1)}$$

where γ_0 is the gyromagnetic ratio, α is the Gilbert damping. $\vec{m}(\vec{r},t) = \vec{M}(\vec{r},t)/M_s$ is the normalized local magnetization to the saturation magnetization (M_s). $\vec{H}_{eff}$ is the effective field, which includes exchange ($\vec{H}_{exc}$), magnetostatic ($\vec{H}_{dmg}$) and Zeeman ($\vec{H}_{dc}$) interactions, along with the Oersted field ($\vec{H}_{Oe}$) generated by the current pulses as discussed above. $\vec{H}_{th}$ is the stochastic thermal field [**S3**]. Further details of all these contributions can be consulted elsewhere [**S1**].

S1.c. Nucleation in the absence of external field

We have preliminary evaluated the minimum amplitude of the current pulse needed to nucleate a reversed domain below the bit line in the absence of external field, $B_{dc} = 0$. Figure S3 shows the temporal evolution of the normalized averaged magnetization along the Ni-NW ($m_x \equiv \langle m_x(\vec{r}) \rangle$) for $B_{dc} = 0$ and for different values of the current pulse ($I_{BL} \neq 0$) flowing through the bit line. Only for current pulses larger than $I_{BL} \approx 275$ mA, which is almost four times larger than the experimental value, the pulse manages to nucleate a reversed domain under the bit line. Interestingly, no matter how large the value of the current or the length of the pulse (Δt_p) is, for $B_{dc} = 0$, the switching of the Ni-NW is never completed. This can be seen in Figure S3(a) because m_x always remains negative and, more graphically, in the snapshots of Figure S3(b). In the absence of external field ($B_{dc} = 0$), the reversed domain, initially flanked by two DWs, ends up collapsing as the time elapses and the magnetization returns to its initial configuration with $m_x \approx -1$.

S1.d. The role of the Gilbert damping and thermal fluctuations for uniform temperature

Fig. 2 of the main text collects representative results for zero temperature and for a perfect Ni-NW reflecting the impossibility of reproducing experimental results of Fig. 1(d). Here we provide further evidence that it is impossible to reproduce the experimental results of Fig. 1(d) by deterministic ($T = 0$) micromagnetic simulations of a perfect Ni-NW. For instance, whereas experimental results indicate that for an external field of $B_{dc} = 100$ mT and a current pulse of $I_{BL} = 65$ mA the switching is deterministically achieved for a pulse length

of the $\Delta t_p \approx 53$ ns (see Fig. 1(d) of the main text), such switching is never achieved for a pulse three times as big, $I_{BL} = 190$ mA, irrespective of the length of the current pulse (see Fig. 2(c)-(e)). Those results were obtained for a perfect Ni-NW at zero temperature.

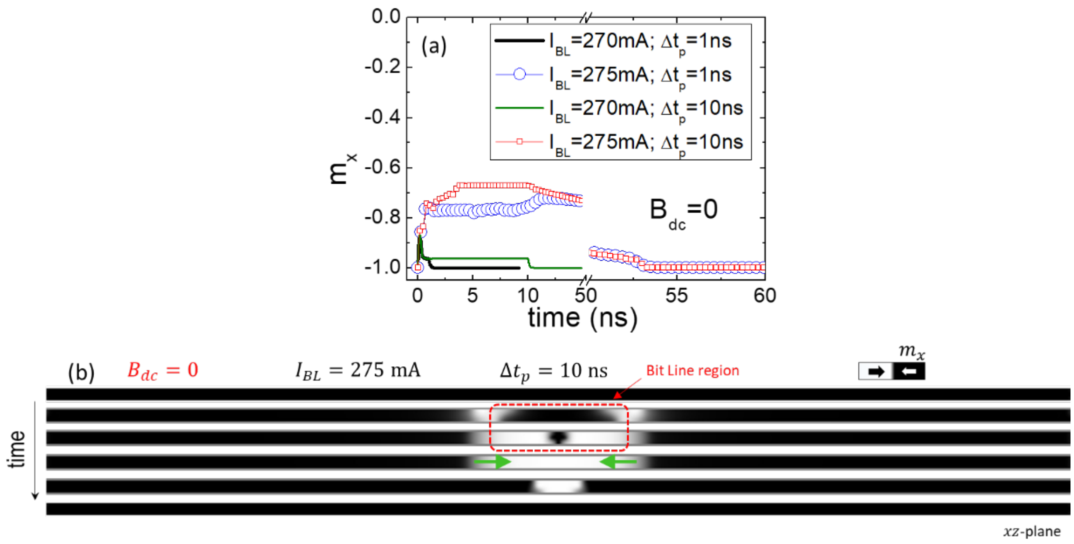

Fig. S3. Domain nucleation by the Oersted field generated by current pulses flowing through the bit line in the absence of external field ($B_{dc} = 0$). (a) Temporal evolution of the average magnetization along the Ni-NW for four combinations of amplitudes (I_{BL}) and durations (Δt_p) of the current pulse. **(b)** Micromagnetic snapshots corresponding to the case $I_{BL} = 275$ mA and $\Delta t_p = 10$ ns.

The role of thermal fluctuations at room temperature ($T = 300$ K) was also evaluated micromagnetically. This was done by taking into account the stochastic thermal field $\vec{H}_{th}$ in the LLG eq. (S1). Note that this thermal field does not include Joule heating effects due to the current pulse flowing through the bit line, and therefore the temperature is uniform along the Ni-NW. We have also studied these processes for perfect Ni-NW and also for a Ni-NW with some edge roughness at room temperature, uniform over the sample. Two different values of the Gilbert damping were also tested, and in each case five different stochastic realizations of the thermal noise were evaluated. Representative results are shown in Fig. S4 for $B_{dc} = 100$ mT, $I_{BL} = 130$ mA and $\Delta t_p = 100$ ns, where the longitudinal component of the magnetization averaged over the Ni-NW ($m_x \equiv \langle m_x \rangle$) remains well below 0, which indicates the absence of nucleation nor switching. For a constant temperature in the Ni-NW,

the experimental results cannot be reproduced, even using longer and larger current pulses than the one used in the experiment (see Fig. 1(d)).

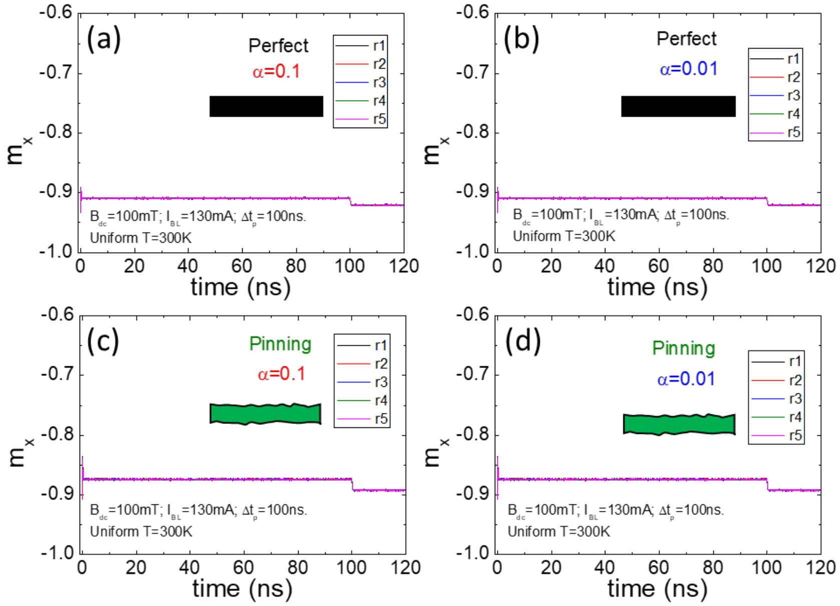

Fig. S4. Micromagnetic results of the temporal evolution of the longitudinal component of the magnetization averaged over the Ni nanowire ($m_x \equiv \langle m_x \rangle$) for uniform room temperature. The current pulse has $I_{BL} = 130$ mA and $\Delta t_{BL} = 100$ ns and the static field is $B_{dc} = 100$ mT. Two different values of the Gilbert damping (α) and both perfect and irregular Ni-NW are considered. Different lines within each graph correspond to different realizations (ri with i:1,…,5) of the thermal noise.

S2. Heat transport characterization by COMSOL simulations

COMSOL simulations [**S4**] solve the heat eq. for the full system shown in Fig. 3(a) of the main text:

$$\frac{\partial T(\vec{r},t)}{\partial t} = \frac{k}{\rho C}\nabla^2 T(\vec{r},t) + \frac{Q(\vec{r},t)}{\rho C} \qquad \text{(S2)}$$

where k is the thermal conductivity (in $\mathrm{W/(K\,m)}$), ρ is the density (in $\mathrm{kg/m^3}$), and C (in $\mathrm{J/(g\,K)}$), is the specific heat capacity of the material. $T = T(\vec{r},t)$ represents the temperature and $Q = Q(\vec{r},t)$ is the heat source, which in this case comes from the Joule heating effect due to the current injected through the bit line (I_{BL}), *i.e.* $Q(\vec{r},t) = J_{BL}^2\,\rho$, with $J_{BL} = I_{BL}/(t_{BL} w_{BL})$, and ρ is the electric conductivity.

The heat generated by Joule effect in the bit line, $Q(\vec{r},t) = J_{BL}^2\,\rho$, is obviously very important in this thermal characterization. Therefore, it is pivotal to obtain the correct value of the resistivity in the Copper and to make sure the current density remains constant during the duration of the current pulse. In Figure S5 we can see the relevant dimensions of the Copper contacts that lead to the Ni-NW. The resistivity of the Copper in the device can be extracted from the resistance of the entire contact (21 Ω) and by calculating the resistance of the different sections, $R_i = \rho\, L_i/A_i$, leaving the resistivity as the unknown. With a simple calculation, the resistivity of the Cu in the bit line results in ρ=4×10^{-8} Ω·m, which is a standard value for thin films, about twice the bulk resistivity. As it can be seen in the thermal analysis (Fig. 3 of the main text), only the narrowest section of the Current line (γ in Figure S5), is going to have a relevant increase in temperature and a change in the resistivity with temperature. This section contributes with $R_\gamma = 2.3\ \Omega$ to the overall 72 Ω load on the pulse generator (22 Ω of the device plus 50 Ω of the high frequency oscilloscope). This implies that only 3.2% of the overall resistive load is heated by the current and only this section would have a resistance increase due to the temperature. Therefore the current density can be assumed constant (J_{BL}~5×10^{11} A/m^2) during the entire length of the current pulse. This was confirmed with the signal measured on the 50 Ω high frequency oscilloscope connected at one end of the bit line. The pulse amplitude remains constant during the entire length of the pulse [**S5**].

The thermal conductivity of the Copper is also a pivotal parameter to determine the correct temperature for a given duration of the current pulse. A common practice is to use the bulk value of the thermal conductivity for Copper, but this can easily lead to a sizable underestimation of the real temperature. Even at the nanoscale, the thermal conductivity is directly related to the electric conductivity (which we just determined for our sample) through the Wiedemann–Franz law $k/\sigma_e = L \cdot T$, with k the thermal conductivity, σ_e the electric conductivity, L the Lorenz number and T the temperature. If possible, the value of the Lorenz number should be selected for the specific thickness of the metal used in the experiment, which is likely different from the Sommerfeld value (2.45×10^{-8} WΩ/deg^2). In our case, we have selected a value of $L = 2.2 \times 10^{-8}$ WΩ/deg^2, which should be adequate for the thickness of Copper we use, according to previous works [**S6**]. This leads to a thermal

conductivity of 165 Wm^{-1}K^{-1}, considerably different to the bulk value (400 Wm^{-1}K^{-1}) but not far from the values found experimentally for Copper films of similar thickness [**S7**].

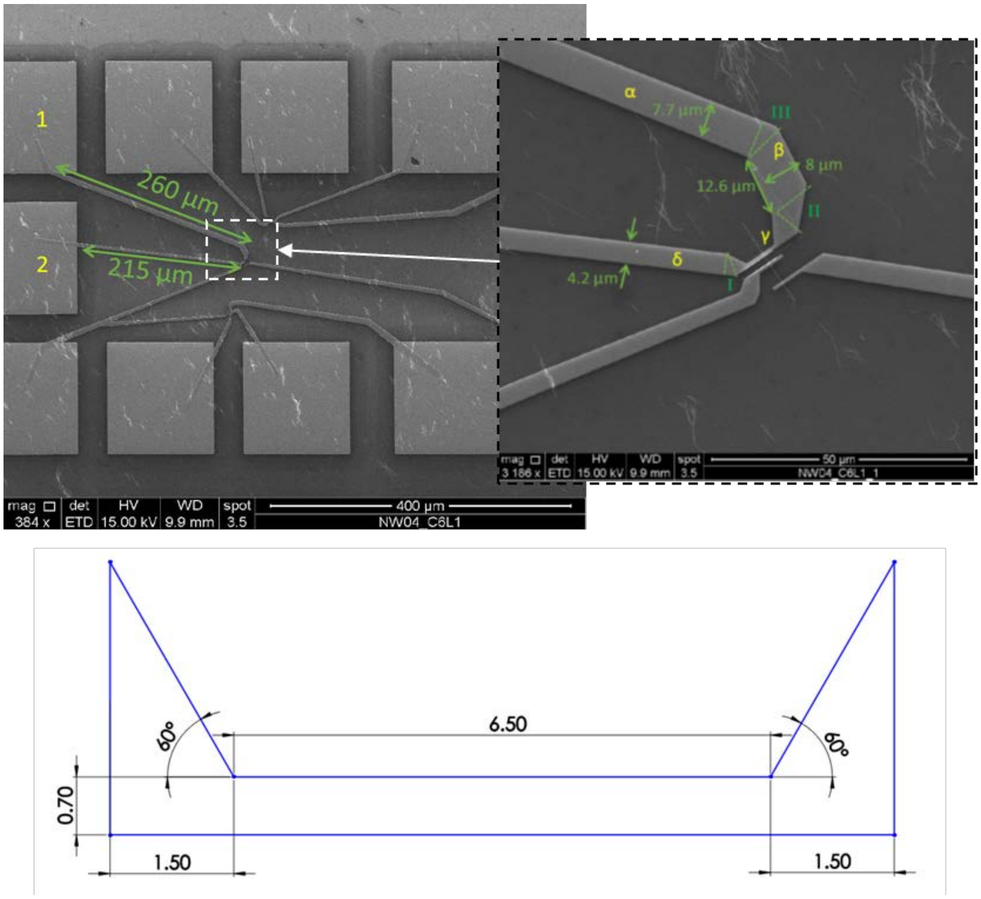

Fig. S5. SEM pictures of the Copper contacts to the Nickel Nanowire. Top figure shows the relevant dimensions of the different sections of the Copper contact lines. The bottom sketch shows the dimensions of the thinnest section where the heat is being generated, which is labelled in the top figure in yellow as γ.

Finally, the thermal conductivity of the Nickel nanowire is likely going to be different to the bulk value due to its nanometric dimensions. Ou *et al*. [**S8**] determined experimentally the thermal conductivity of a Ni-NW of similar dimensions. At Room Temperature they obtained a value of 22 Wm^{-1}K^{-1}. We assumed this value to be constant with temperature, which is not, but we have checked that even a 30% difference in the value of the thermal conductivity does not have a significant effect in the final result of the simulation. Note that the thermal conductivity for the Ni-NW is important for the process of heat transmission along the Ni-NW, but it is almost irrelevant when obtaining the temperature underneath the bit line for a

given pulse. This can be easily understood in terms of the large heat capacity of the Copper bit line in comparison to the much smaller heat capacity in the considerably smaller volume of the Ni-NW.

S3. Details of micromagnetic simulations with Joule heating

COMSOL simulations presented in the main text (Fig. 2 of the main text, and Sec. S2 of this Supplementary Material) clearly indicate that the current pulse flowing through the bit line generates, not only an Oersted magnetic field, but also a significant local heat on the Ni-NW. This local heat distribution evolves in time during and after the current pulse along the bit line, and consequently the temperature becomes space- and time-dependent along the Ni-NW. Therefore, any realistic description of the experimental switching process requires to take into account the ***magnetization dynamics coupled to the heat transport*** under both current pulses $(I_{BL}, \Delta t_p)$ and the static magnetic field $\vec{B}_{dc}$. Here we present the details of the micromagnetic model which allows us to evaluate such magneto-thermal dynamics.

Since the nanowire reaches temperatures near or even above the Curie temperature of the Ni ($T_C = 631$ K), we cannot use the LLG eq. (S1), which is only valid for uniform temperatures well below the Curie threshold ($T \neq f(\vec{r}, t)$ and $T \ll T_C$). Therefore, the ***magnetization dynamics*** is studied here using the Landau-Lifshitz-Bloch eq. [S9,S10]

$$\begin{aligned}\frac{d\vec{m}(\vec{r},t)}{dt} &= -\gamma_0'\vec{m}\times\vec{H}_{eff} - \gamma_0'\frac{\alpha_\perp}{m^2}\left[\vec{m}\times\left(\vec{m}\times\left(\vec{H}_{eff}+\vec{H}_{th}^{\perp}\right)\right)\right] \\ &\quad + \gamma_0'\frac{\alpha_\parallel}{m^2}\left(\vec{m}\cdot\vec{H}_{eff}\right)\vec{m} + \vec{H}_{th}^{\parallel}\end{aligned} \tag{S3}$$

where $\gamma_0' = \gamma_0/(1+\lambda^2)$, with $\lambda \equiv \alpha$ being the conventional Gilbert damping. Apart from the standard precessional and damping terms (1st and 2nd terms on the RHS of eq. (S3)), the LLB eq. (S3) includes an additional longitudinal relaxation term (3rd term in eq. (S3)) which describes the relaxation of the module of $\vec{m}$ towards its equilibrium value $m_e(T)$. $m_e(T)$ represents the normalized equilibrium magnetization at each local temperature, which we calculate by Langevin function, namely [S10,S11] (see Fig. S7(a)). A characteristic feature of the LLB eq. (S3) is precisely that, contrary to the LLG eq. (S1), $\vec{m}(\vec{r}, t)$ is not unitary but its module varies depending on the local temperature $T(\vec{r}, t)$. Note that this longitudinal

relaxation is neglected in the LLG formalism since, at $T = 300$ K, it is much faster than the transverse relaxation. However, it becomes particularly relevant at T close to T_C, where the longitudinal and the transverse relaxation times are comparable. $\alpha_\perp$ and $\alpha_\parallel$ are the transverse and the longitudinal damping parameters, respectively. They depend on the temperature [S13,S14,S15] as $\alpha_\perp = \alpha\left(1 - \frac{T}{3T_C}\right)$ and $\alpha_\parallel = \alpha\left(\frac{2T}{3T_C}\right)$ for $T < T_C$, while $\alpha_\perp = \alpha_\parallel = \alpha\left(\frac{2T}{3T_C}\right)$ for $T > T_C$. Note that in the limiting case of zero temperature, $\alpha_\perp$ reduces to the conventional Gilbert damping $\alpha_\perp = \alpha$, and $\alpha_\parallel = 0$. The effective field $\vec{H}_{eff}$ in eq. (S3) is given by [S13,S14,S15]

$$\vec{H}_{eff} = \vec{H}_{exch} + \vec{H}_{dmg} + \vec{H}_{dc} + \vec{H}_{Oe} + \vec{H}_m \tag{S4}$$

where $\vec{H}_{dmg}$, $\vec{H}_{dc}$ and $\vec{H}_{Oe}$ are the magnetostatic field, the external field and the Oersted field, respectively. The exchange field now reads as [S15]

$$\vec{H}_{exch} = \frac{2A(T)}{m_e^2 M_s^0}\nabla^2\vec{m} \tag{S5}$$

where $A(T)$ is the temperature-dependent exchange constant. Here we follow the same assumption adopted in [S16,S17], where $A(T)$ scales with T as $A(T) = A^0 m_e^2(T)$. M_s^0 and A^0 represent the saturation magnetization and the exchange constant at $T = 0$. The last term in eq. (S3), $\vec{H}_m$, which is not present in the LLG eq. (S1), is [S13,S14,S15]

$$\vec{H}_m = \begin{cases} \dfrac{1}{2\chi_\parallel}\left(1 - \dfrac{m^2}{m_e^2}\right)\vec{m}, & T < T_C \\[2ex] -\dfrac{1}{\chi_\parallel}\left(1 + \dfrac{3}{5}\dfrac{T_C m^2}{(T - T_C)}\right)\vec{m}, & T > T_C \end{cases} \tag{S6}$$

where $\chi_\parallel$ is the so-called longitudinal susceptibility. See [S9,S10] and references therein for further details. Finally, the stochastic fields $\vec{H}_{th}^{\perp,\parallel}$ take into account longitudinal and transverse fluctuations. They are considered to have white noise properties with the following correlators [S18]:

$$\langle \vec{H}^{\perp}_{th,i}(\vec{r},t)\vec{H}^{\perp}_{th,j}(\vec{r}',t')\rangle = \frac{2k_B T(\alpha_\perp - \alpha_\parallel)}{\gamma_0 \mu_0 M_s^0 V \alpha_\perp^2}\delta_{ij}\delta(\vec{r}-\vec{r}')\delta(t-t') \tag{S7}$$

$$\langle \vec{H}^{\parallel}_{th,i}(\vec{r},t)\vec{H}^{\parallel}_{th,j}(\vec{r}',t')\rangle = \frac{2k_B T\alpha_\parallel}{\mu_0 M_s^0 V}\delta_{ij}\delta(\vec{r}-\vec{r}')\delta(t-t') \tag{S8}$$

where i,j: $\{x,y,z\}$ represents the Cartesian coordinates indexes and V is the volume of the computational cell. Again, further details of the LLB eq. (S3) can be consulted in [S9,S10] and references therein.

To account for the ***Joule heating***, the LLB eq. (S3) has to be numerically solved coupled to the heat transport eq, which was already introduced Sec. 2 of this Supplementary Information. Since the inclusion of the complete system under study (bit line, Ni-NW, contacts C and D, substrate and surrounding media) into the micromagnetic code would be prohibitive from a computational point of view, we adopted a phenomenological model by solving the following phenomenological heat equation along the Ni-NW [S9],

$$\frac{\partial T(\vec{r},t)}{\partial t} = \frac{k}{\rho C}\nabla^2 T(\vec{r},t) + \frac{Q(\vec{r},t)}{\rho C} - D(\vec{r},t) \tag{S9}$$

where the last term in eq. (S9) is a dissipation term that takes into account the heating (and the cooling) of the Ni-NW due to the bit line current pulse, the substrate, the contact line C and the surrounding ambient. Fig. S6(a) defines the geometry for the heat eq. (S9). In order to fit COMSOL results (Fig. 3 in main text), this dissipation term can be expressed as $D(\vec{r},t) = (T(\vec{r},t) - T_0)/\tau$. $T_0 = 300$ K represents the temperature of the surrounding ambient (including the contacts), and the parameter τ is the characteristic time that represents the rate at which the heat is exchanged between Ni-NW and the surroundings. This parameter τ is space -dependent, $\tau = \tau(\vec{r})$: $\tau = 82$ ns in all points of the Ni-NW, except for the conducting line C (see Fig. 1(b) in the main text) which is acting as a sink for the heat of the Ni-NW. In the region of the Ni-NW below the contact C ($d - \frac{w_{BL}}{2} \leq x \leq d + \frac{w_{BL}}{2}$) the characteristic time is $\tau = 1$ ns. Note that a relatively high (low) characteristic time τ means a poor (good) heat dissipation.

The heat dynamics predicted by this phenomenological heat model (eq. (S9)) is shown in Fig. S6(b)-(d). The temporal evolution of the central temperature predicted by the heat eq. (S9) looks quantitatively very similar to the one obtained by COMSOL simulations of the full systems (Fig. S6(b)). As it can be seen in Fig. S6(c)-(d), a good agreement with COMSOL simulation of the entire systems is achieved (see Fig. 3 in the main text) for the space and temporal evolution of the temperature. These results validate the use of the 1D heat eq. (S9) to be solved at the same time with the LLB eq. (S3).

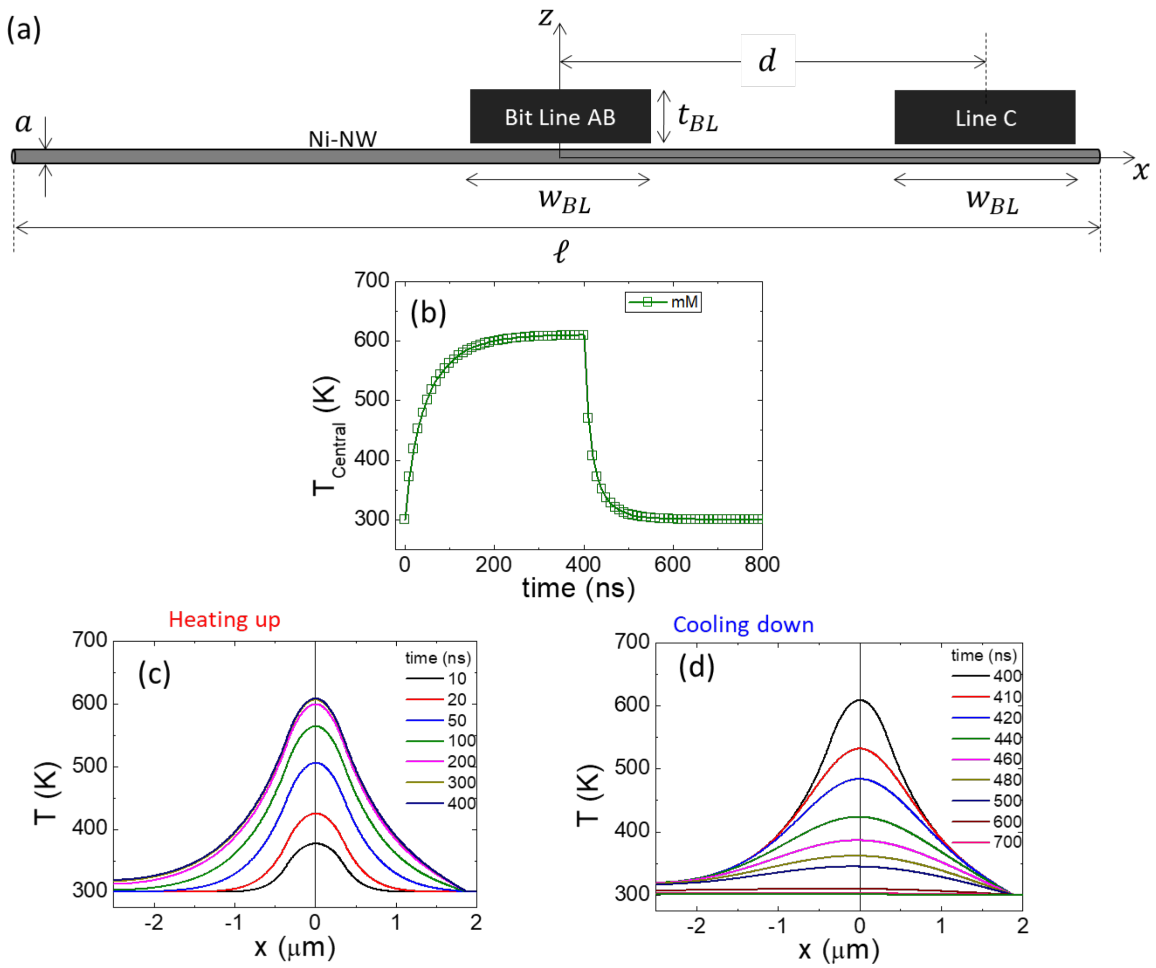

Fig. S6. Validation of the Phenomenological heat transport as predicted by heat eq. (S9). (a) Geometry of the phenomenologycal heat problem defining the bit line (A-B in Fig. 1), the conducting line (line C in Fig. 1) and the Ni-NW. (b) Comparison of the central temperature deduced from COMSOL simulation and the prediction of the heat (eq. (S9)). (c) and (d) depict the temporal evolution of the temperature along the Ni-NW as predicted by the heat eq. (S9).

S4. Micromagnetic results with Joule heating

Here we present additional micromagnetic results to complement the ones presented in Fig. 4 of the main text, and to strengthen our interpretation. To evaluate the magnetization dynamics along the Ni-NW as coupled to the heat transport, we solved numerically the LLB eq. (S3) and the heat eq. (S9) simultaneously using a Heun' method. Representative results were shown in Fig. 4 of the main text. Note that within this magneto-thermal framework, the magnetization dynamics, LLB eq. (S3), is coupled to the temperature dynamics eq. (S9), both through the stochastic thermal fields ($\vec{H}_{th}^{\perp,\parallel}$) and the temperature dependence of the magnetic parameters ($M_s = M_s(T)$, $A = A(T)$, $\alpha_\perp = \alpha_\perp(T)$ $\alpha_\parallel = \alpha_\parallel(T)$).

As already claimed in the main text, the key physics of the experimental observations (dependence of the switching threshold pulse length, Δt_p, on the dc magnetic field B_{dc}, Fig. 1(c) in the main text) is the reduction of the saturation magnetization with temperature, $M_s = M_s(T)$. In Fig. S7(a), we plot the temperature dependence of the spontaneous magnetization, given by the Langevin function, which, as it was mentioned, is taken into account within the LLB formalism, eq. (S3). In Fig. S7(b) we plot again the temporal evolution of the central temperature (just below the bit line) as predicted by COMSOL and the 1D heat eq. (see Fig. 6(b)), and in Fig. S7(c) we show the temporal evolution of M_s in the same central point during the injection of the current pulse along the bit line ($I_{BL} = 65$ mA and $\Delta t_p = 400$ ns).

In Fig. 4 of the main text, we plotted the micromagnetic snapshots for an external field of $B_{dc} = 100$ mT and two different pulse lengths: $\Delta t_p = 51$ ns and $\Delta t_p = 53$ ns, which results in no-switching and switching respectively. The amplitude of the current pulse is the same as in the experiment ($I_{BL} = 65$ mA). We have also extracted spatial profiles of the local temperature $T = T(x)$ (top graphs) and the longitudinal magnetization component $m_x(x)$ (bottom graphs) along the Ni-NW axis (x-axis) at some representative instants during and after the current pulse for these representative cases. The results are shown in Fig. S8. Movies of this magneto-thermal dynamics are also provided as Supplementary Material: see Sec. S5 at the end of the present document.

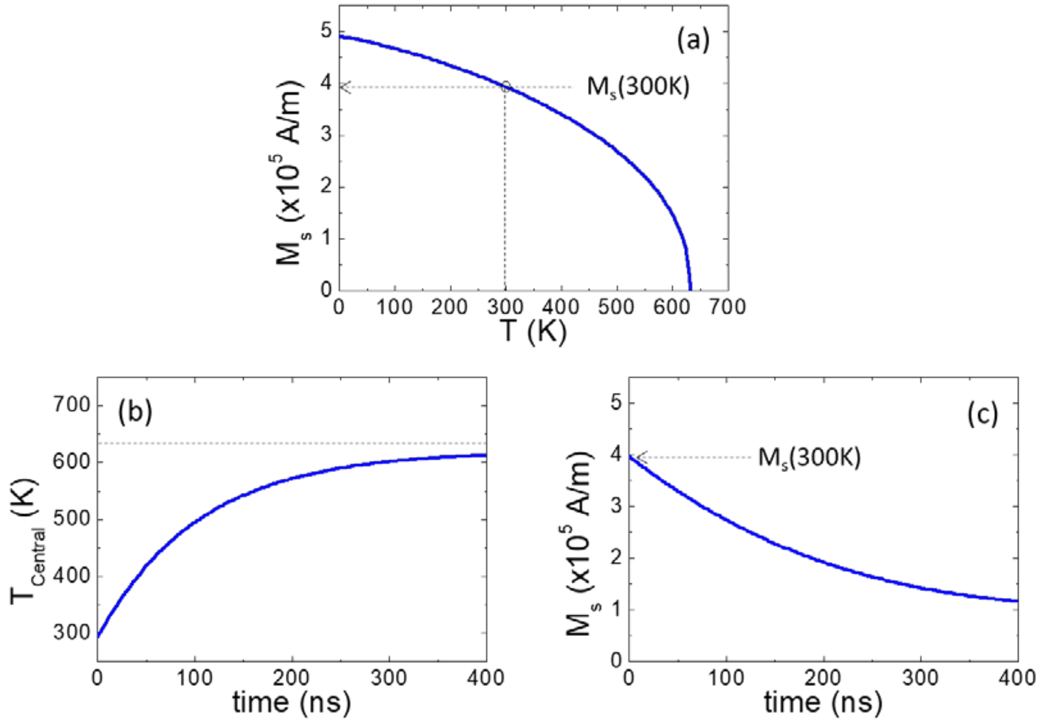

Fig. S7. (a) Saturation magnetization (M_s) as a function of the temperature (T) considered in the micromagnetic simulations which coupled the LLB eq. (S3) and the heat eq. (S9). This curve is usually referred as the Langevin function. (b) Temporal evolution of the central temperature in the Ni-NW(just below the bit line) during a current pulse of the $\Delta t_p = 400$ ns (COMSOL result). The horizontal dashed line indicates the Curie temperature. (c) Temporal dependence of M_s in the central point (just below the bit line) under the current pulse with $I_{BL} = 65$ mA and $\Delta t_p = 400$ ns.

In order to further support our interpretation, we have performed additional micromagnetic simulations solving the LLB eq. (S3) and the heat eq. (S9) under different conditions. The amplitude of the current pulse is fixed to its experimental value ($I_{BL} = 65$ mA). The phase diagram depends on the local heat of the Ni-NW, $Q(\vec{r}, t)$, which is the only input parameter in our magneto-thermal modeling. In Fig. S9(a) we plot the micromagnetic results for three different values of $Q(\vec{r}, t)$ along with the experimental ones. A remarkable quantitative agreement is achieved for $Q = 3.75 \times 19^{16}$ W/m^3. For larger values ($Q = 5.0 \times 19^{16}$ W/m^3) the critical switching phase diagram indicates an almost linear dependence of critical pulse duration Δt_p on B_{SW}. On the contrary, for smaller values ($Q = 2.4 \times 19^{16}$ W/m^3), the linear regime is restricted to the range 130 mT $\lesssim B_{SW} < 145$ mT and, for smaller fields, Δt_p increases abruptly. These results were obtained with $\alpha = 0.1$ and

neglecting thermal fluctuations (no thermal noise, $\vec{H}_{th}^{\perp,\parallel} = 0$). In Fig. S9(b) we evaluate the role of the Oersted field ($\vec{B}_{Oe}$) by comparing the micromagnetic results with ($\vec{B}_{Oe} \neq 0$) and without this field ($\vec{B}_{Oe}$=0) when evaluating the phase diagram. Again, these results were obtained with $\alpha = 0.1$ and without thermal noise ($\vec{H}_{th}^{\perp,\parallel} = 0$). As expected the influence of the Oersted field increases as the external field B_{dc} decreases. Note that the amplitude $\vec{B}_{Oe}$ is fixed by the current flowing through the bit line, and that the maximum amplitude of the longitudinal component this field ($B_{Oe,x}$, see Fig. S2(a)) is significantly smaller than the switching field in the absence of current pulse ($\cong 142$ mT). These results reinforce the pivotal role of the local heat as the external field is reduced, which is clearly much more relevant than the Oersted field.

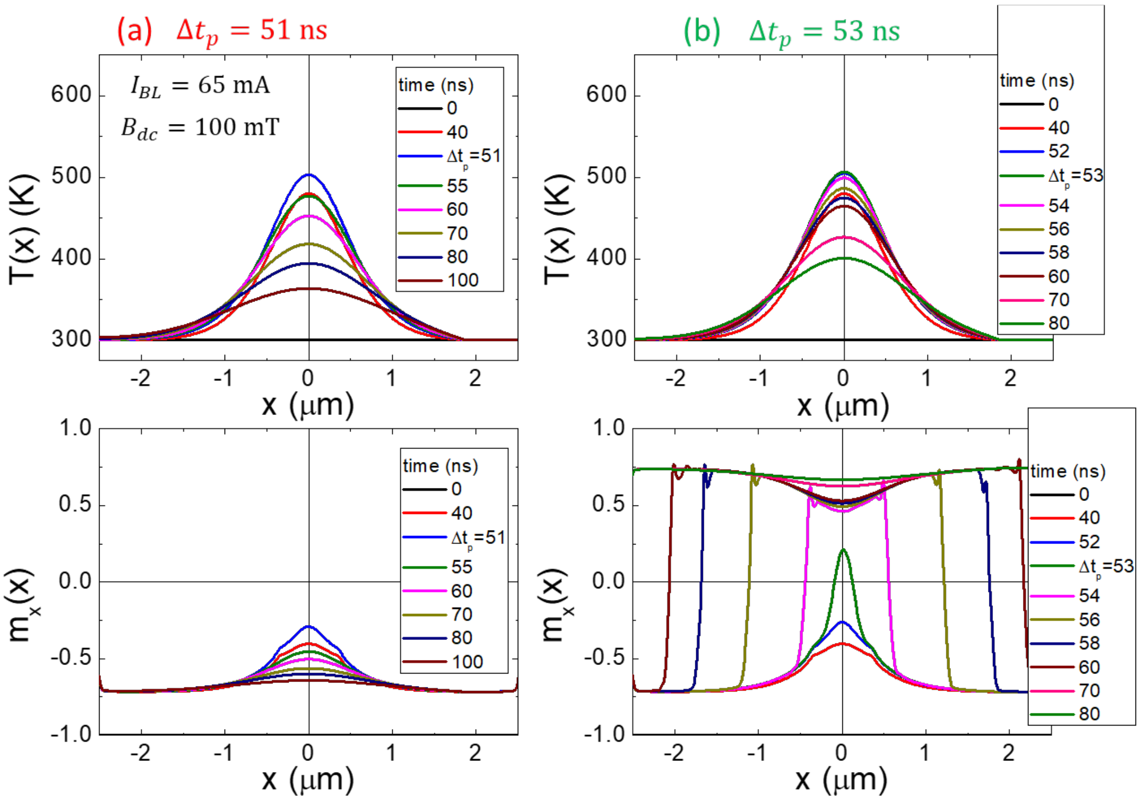

Fig. S8. Space and time evolution of the temperature $T(x,t)$ and the longitudinal magnetization component $m_x(x,t)$ along the Ni-NW axis, as predicted by the micromagnetic model which solves the LLB eq. (S3) and the heat eq. (S9) simultaneously, in the absence of thermal noise. The amplitude of the current pulse and the external magnetic field are $I_{BL} = 65$ mA and $B_{dc} = 100$ mT. Two duration of the current pulses are shown: (a) $\Delta t_p = 51$ ns (No-Switching), and (b) $\Delta t_p = 53$ ns (Switching).

We have also evaluated the phase diagram for the different values of the Gilbert damping (α). The results presented in Fig. S9(c), clearly indicate that the damping is not playing any relevant role in these heat controlled process.

Finally, in order to highlight the deterministic nature of this thermal switching we have also taken into account thermal fluctuations in the form given by eqs. (S7)-(S8). The results with thermal noise are presented in Fig. S9(d) (orange curve, $\vec{H}^{\perp}_{th,i}, \vec{H}^{\parallel}_{th,i} \neq 0$) and compared to the ones obtained without thermal noise (blue curve, $\vec{H}^{\perp}_{th,i}, \vec{H}^{\parallel}_{th,i} = 0$). Thermal fluctuations marginally anticipate the switching phase diagram with respect to the pure deterministic modelling, which further supports our claims. In summary, all these micromagnetic results give a very robust interpretation of the experiment and prove that the switching described in the experiment is essentially controlled by the local heat delivered to the Ni-NW from the current pulse flowing through the bit line.

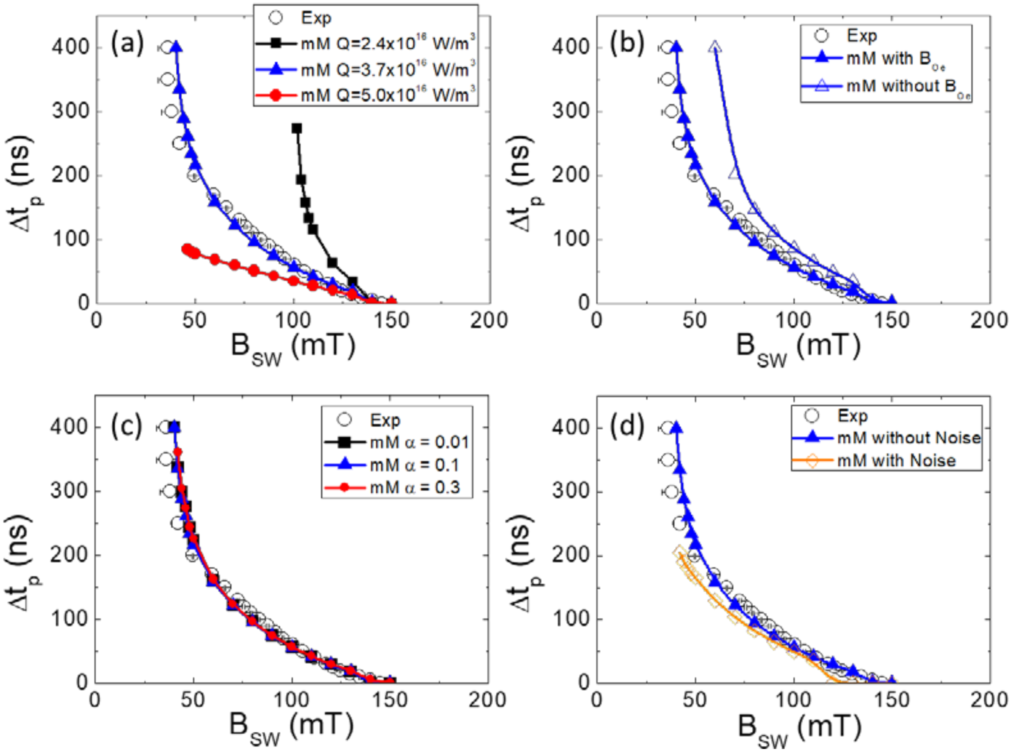

Fig. S9. Micromagnetic phase diagrams obtained under different conditions: (a) Different input values Q_0. **(b)** The role of the Oersted field ($\vec{B}_{Oe}$). **(c)** The role of the Gilbert damping (α). For $\alpha = 0.3$, the results were computed from $B_{SW} = 150$ mT up $B_{SW} = 50$ mT. **(d)** The role of stochastic thermal fluctuations ($\vec{H}^{\perp}_{th,i}, \vec{H}^{\parallel}_{th,i} \neq 0$). Except the contrary is indicated the Gilbert damping is $\alpha = 0.1$, the heat input $Q_0 = 3.75 \times 10^{16}$ W/m^3, and no thermal noise is taken into account ($\vec{H}^{\perp}_{th,i} = \vec{H}^{\parallel}_{th,i} = 0$).

S5. Supporting Material: videos of the magneto-thermal dynamics

Movies showing the temporal evolution of the temperature $T(x,t)$ and the longitudinal magnetization component $m_x(x,t)$ along the Ni-NW axis are provided:

v1_Bdc100mT_tp51ns.mp4: No Switching for $B_{dc} = 100$ mT, $I_{BL} = 65$ mA, and $\Delta t_p = 51$ ns.

v2_Bdc100mT_tp53ns.mp4: Switching for $B_{dc} = 100$ mT, $I_{BL} = 65$ mA, and $\Delta t_p = 53$ ns.

Within these videos we show:

- $T(x,t)$ and $m_x(x,t)$: top-left graph.
- $T_{Central}$ and $\langle m_x \rangle$ *vs* time: top-right graph.
- Temporal evolution of T and m_x in the central xz- plane: bottom movies.

S6. Details of macrospin model

As mentioned in the main text, the macrospin model assumes that the hot region below the bit line is uniformly magnetized and that it reverses independently of the rest of the nanowire. Based on these assumptions the energy density of this hot region is given by

$$\epsilon = -K_{sh}\cos^2\varphi - M_S(T)[B_{dc}\cos(\varphi - \phi_B) + B_{Oe}] \qquad \text{(S10)}$$

where φ and ϕ_B are the angle of the magnetization and the applied field with the nanowire axis respectively (Figure 5(a)), whereas the Oersted field created by the current pulse, B_{Oe}, is assumed to be uniform along the x direction. The shape anisotropy term can be expressed as a function of the magnetostatic energy as follows,

$$K_{sh}(T) = \frac{1}{2}\mu_0 M_S^2(T) N_\perp - \frac{1}{2}\mu_0 N_\parallel\,(M_s(T_0) - M_s(T))M_S(T) \qquad \text{(S11)}$$

where $N_\perp$ and $N_\parallel$ are the demagnetizing factors perpendicular to and along the nanowire axis respectively [**13**] and T_0 is room temperature. Note that the second term accounts for the magnetostatic energy arising from the discontinuity in the value of the magnetization

between the hot section and the rest of the Ni-NW as shown in Figure 5(a). The switching field is given by

$$B_{SW}(T(t)) = \frac{1}{(\sin^{2/3}\phi_B + \cos^{2/3}\phi_B)^{3/2}} \frac{2K_{sh}(T(t))}{M_s(T(t))} \tag{S12}$$

This expression makes the switching field a monotonically decreasing function of temperature through the temperature dependent saturation magnetization $M_s(T)$. Thus, for each value of the external applied field B_{dc}, we compute the instant in time that makes $B_{SW}(T(t)) = B_{dc}$. The results are shown in Fig. 4(d) together with experimental data and micromagnetic simulations. To obtain them we assumed that $M_s(T)$ follows Langevin's law with $T_C = 631\ \mathrm{K}$ and $M_s(0) = 4.9 \times 10^5\ \mathrm{A/m}$ and that temperature evolves in time as derived from COMSOL thermal characterization underneath the center of the bit line ($x = 0$), namely $T(t) = 620\ \mathrm{K} - 327\ \mathrm{K}\exp(-t/104\mathrm{ns})$. We also assumed $B_{Oe} = 11\ \mathrm{mT}$, which is roughly the maximum value of the longitudinal component of the Oersted field (see Fig. S2(a) for $I_{BL} = 65\ \mathrm{mA}$), and $\frac{N_{\parallel}}{N_{\perp}} = 0.1$, which corresponds to a reversed region of the same length than the bit line (700 nm).

S7. Example of the measuring routine for the nanowire switching

Figure S10, shows several examples of the automatic routine used to measure the switching of the Ni-NW. In this example, we selected the measurements for long pulses, where the switching field is of the order of 40 mT. This is the only field were we detected stochastic (rather than deterministic) switching, as the temperature in the Ni-NW is getting very close to its Curie Temperature. The automatic routine cycles the field and searches for the AMR jump that, for no pulse, is detected at 142 mT. If the AMR is still there (marked with red circles in Fig. S10), the current pulse (and its associated Joule heating) did not achieve the switching for that particular external field. The three selected cases show how at low fields (32.5 mT) there are only 2 switching events, while for the correct switching field (40 mT), only one event does not achieve switching.

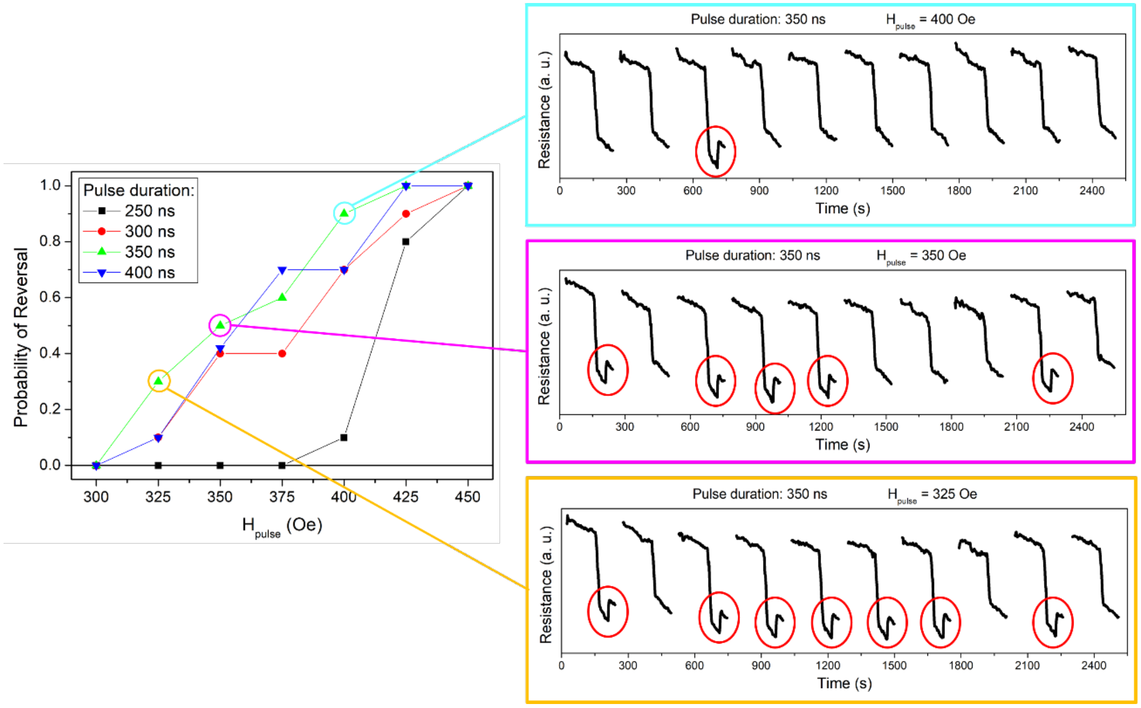

Fig. S10. Stochastic switching close to Curie Temperature. Different sequences to measure the reliability of the switching for long current pulses and a switching field around 40 mT. The sharp changes in resistance marked in red correspond to the switching of the magnetization assisted by the external applied magnetic field. These are thus accounted as events were the applied pulse did not switch the magnetization of the nanowire.

Supplementary references

[S1] A. Vansteenkiste, Jonathan Leliaert, Mykola Dvornik, Mathias Helsen, Felipe Garcia-Sanchez, and Bartel Van Waeyenberge. *The design and verification of MuMax3*, AIP Advances 4, 107133 (2014).

[S2] O. Alejos, V. Raposo, L. Sanchez-Tejerina and E. Martinez. *Efficient and controlled domain wall nucleation for magnetic shift register*. Scientific Reports, 7, 11090, (2017).

[S3] E. Martinez, L. Lopez-Diaz, L. Torres, C. Tristan, and O. Alejos. *Thermal effects in domain wall motion: Micromagnetic simulations and analytical model*. Phys. Rev. B 75, 174409 (2007).

[S4] COMSOL Multiphysics. www.comsol.com.

[S5] E. Ramos, C. Lopez, M. Munoz, and J. L. Prieto, Phys. *Joule heating in ferromagnetic nanostripes with a notch*. Rev. B 91, 214404 (2015).

[S6] Avery, A. D., Mason, S. J., Bassett, D., Wesenberg, D. & Zink, B. L. *Thermal and electrical conductivity of approximately 100-nm permalloy, Ni, Co, Al, and Cu films and examination of the Wiedemann-Franz Law*. *Phys. Rev. B* **92**, 214410 (2015).

[S7] Nath, P. & Chopra, K.L. *Thermal conductivity of Copper films*. Thin Solid Films, 20 53 (1974).

[S8] Ou, M.N. et al. *Electrical and thermal transport in single nickel nanowire*. Appl. Phys. Lett. 92, 063101 (2008).

[S9] S. Moretti *et al*, *Influence of Joule heating on current induced domain wall depinning*, Journal of applied Physics, 119, 213902 (2016).

[S10] S. Moretti et al. *Domain wall motion by localized temperature gradients*. Phys. Rev. B 95, 064419 (2017).

[S11] G. Bertotti, *Hysteresis in Magnetism* (Academic Press, 1998).

[S12] P. E. Mijnarends, S. Sahrakorpi, M. Lindroos, and A. Bansil. *Angle-resolved photoemission spectra, electronic structure, and spin-dependent scattering in* $Ni_{1-x}Fe_x$ *Permalloys*. Phys. Rev. B 65, 075106 (2002).

[S13] D. A. Garanin. *Fokker-Planck and Landau-Lifshitz-Bloch equations for classical ferromagnets*. Phys. Rev. B 55, 3050 (1997).

[S14] O. Chubykalo-Fesenko, U. Nowak, R. Chantrell, and D. Garanin. *Dynamic approach for micromagnetics close to the Curie temperature*. Phys. Rev. B 74, 094436 (2006).

[S15] N. Kazantseva, D. Hinzke, U. Nowak, R. Chantrell, U. Atxitia, and O. Chubykalo-Fesenko. *Towards multiscale modeling of magnetic materials: Simulations of FePt*. Phys. Rev. B 77, 184428 (2008).

[S16] U. Atxitia, O. Chubykalo-Fesenko, N. Kazantseva, D. Hinzke, U. Nowak, and R. W. Chantrell. *Micromagnetic modeling of laser-induced magnetization dynamics using the Landau-Lifshitz-Bloch equation*. Appl. Phys. Lett. 91, 232507 (2007).

[S17] A. J. Ramsay, P. E. Roy, J. A. Haigh, R. M. Otxoa, A. C. Irvine, T. Janda, R. P. Campion, B. L. Gallagher, and J. Wunderlich. *Optical Spin-Transfer-Torque-Driven Domain-Wall Motion in a Ferromagnetic Semiconductor*. Phys. Rev. Lett. 114, 067202 (2015).

[S18] R. F. L. Evans, D. Hinzke, U. Atxitia, U. Nowak, R. W. Chantrell, and O. Chubykalo-Fesenko. *Stochastic form of the Landau-Lifshitz-Bloch equation*. Phys. Rev. B 85, 014433 (2012).
